# Missouri State Veterinary Medical Association

**Published:** 1895-12

**Authors:** T. F. Arnold

**Affiliations:** Secretary


### SOCIETY PROCEEDINGS.

### MISSOURI STATE VETERINARY MEDICAL ASSOCIATION.

THE second annual meeting was held at Moberly, September 4, 1895, in the City Hall.

The meeting was called to order at 9 A.M. The Mayor, W. P. Cave, extended a hearty welcome to all, which was responded to by Dr. Charles Ellis, of St. Louis. Minutes of the last meeting were read and approved. On roll-call the following members responded to their names: Drs. T. J. Turner (President), T. F. Arnold (Vice-President), H. B. Platt (Secretary-Treasurer), L. M. Klutts, L. E. Booth, J. H. Wattles, S. E. Phillips, H. F. James, A. J. Hammerstein, Charles Ellis, T. B. Howard, and G. T. Netherton.

The following applicants were proposed for membership, approved by the committee, and elected: George W. Vernon, Dexter; J. H. Cock, Knob Noster; B. F. Kaupp, Nevada; W. F. King, Ladue; R. A. Ramsey, Mexico; F. W. O'Brien, Hannibal; W. E. Martin, Perry; C. O. Netherton, Carrollton; Horace Bradley, Windsor; H. B. Goode, Lexington; G. W. Dickey and J. T. Crosby, St. Louis; and A. O. Longnecker, Paris.

The Constitution and By-laws, as reported, were then read and adopted. The Code of Ethics was then read and adopted.

It was then moved and seconded that a committee be appointed to draft suitable resolutions on the death of one of our worthy members—P. F. Kiernan, of Jefferson Barracks; carried. The Vice-President, having been called to the chair, appointed the following committee: Drs. J. H. Wattles, L. M. Klutts, and J. E. Phillips, who subsequently reported the subjoined resolutions: "Whereas, it has pleased Almighty God to remove from our midst Dr. P. F. Kiernan, a worthy member of this Association, be it resolved that we tender to his family in their great bereavement our heartfelt sympathy; and that a copy of these resolutions be spread upon the minutes of our Association and furnished the veterinary journals for publication."

The following were elected officers for the ensuing year: T. J. Turner, President, Columbia; J. H. Wattles, Vice-President, Kansas City; T. F. Arnold, Secretary and Treasurer, Lewiston.

Moved and seconded to adjourn and meet in Mexico next September; carried.

T. F. ARNOLD, V. S.,
Secretary.

